# Case of Gun-Shot Wound, with Fracture into the Elbow-Joint—Read before Students’ Medical Society

**Published:** 1864-07

**Authors:** E. N. B. Smith


ART. III.—*Case of Gun-Shot Wound, with fracture into the Elbow-Joint—Read before Students' Medical Society.* By E. N. B. Smith.

John Brunner, Co. B, 49th Regiment, N. Y. Vols., arrived in this city, Thursday, May 19th, had been wounded at the battle of the "Wilderness," May 5th, two weeks previous to his arrival. Dr. Long conducted him to the office of Dr. Miner, who, by request examined the wound. The ball, a Minié, had entered the fore-arm about 1½ inches below the olecranon process.

May 20th, Dr. Miner, accompanied by Drs. Long, Lothrop, Stork, Brown and medical students, visited the patient at his home for the purpose of making any operation the appearance of the joint might indicate. The arm was badly swollen from the tips of the fingers to the shoulder, and, judging from external appearance, the joint was so badly broken up that much doubt was entertained of saving the arm. Dr. Miner thought best to lay open the wound and find out its internal condition, which was done by making an incision about 4 inches in length directly over the external wound. The ball had broken off the olecranon process, shattered the ulna to a distance of 2½ or 3 inches below its point of entrance, and also fractured the inner condyle of the humerus, not touching the radius, which retained its normal position. Not finding the joint as badly comminuted as he expected, he removed all the pieces of bone and the ball; cleansed the wound, drew it together, and applied a cold compress; gave morphia sulph ½ gr. to quiet pain when necessary.

The principal point of interest in this case is the effort made to save the arm, whereas eight or ten years ago it was thought impossible to save a limb that had a joint opened or badly injured—conservative surgery in such a case as this was considered out of the question.

It will be observed that the results of the case are as yet undetermined, and possibly the patient may lose his arm, or even his life. Whatever may be the termination the case is an interesting one; and when the result is known will contribute one case towards establishing what course it is safe and best to pursue under similar circumstances.*

* This patient is rapidly recovering, and the motions of the joint are retained as yet in remarkable degree.

